# Sociopolitical Factors and Mental Health Following the Turkey-Syria Earthquake

**DOI:** 10.1001/jamanetworkopen.2024.11413

**Published:** 2024-05-15

**Authors:** Wai Kai Hou, Tiffany Junchen Tao, Crystal Jingru Li, Evon Lam Wong, Aysuhan Tuba Saral, Huinan Liu, Sandro Galea

**Affiliations:** 1 Centre for Psychosocial Health, The Education University of Hong Kong, Hong Kong SAR, China; 2 Department of Psychology, The Education University of Hong Kong, Hong Kong SAR, China; 3 Department of Psychological Science, University of California, Irvine; 4 Department of International Education, The Education University of Hong Kong, Hong Kong SAR, China; 5 Department of Special Education and Counselling, The Education University of Hong Kong, Hong Kong SAR, China; 6 School of Public Health, Boston University, Boston, Massachusetts

## Abstract

This survey study evaluates the association between sociopolitical factors and mental health following the 2023 Turkey-Syria earthquake.

## Introduction

Large-scale natural disasters like the 7.8-magnitude Turkey-Syria earthquake on February 6, 2023, can be followed by substantial mental health consequences.^1^ The impact of these events can be modified by ongoing stressors of financial strain,^2^ political turmoil, and civil unrest.^3,4^ This study aims to investigate the associations between earthquake exposure and subsequent probable psychiatric conditions and whether and how these associations differed across socioeconomic status, assets, and political concerns surrounding the Turkish presidential election in May 2023.

## Methods

A nationally representative Turkish sample was recruited through TGM Research’s internet panel. Participants completed an online survey from September to October 2023 (75.9% response rate). This study followed the American Association for Public Opinion Research (AAPOR) and Strengthening the Reporting of Observational Studies in Epidemiology (STROBE) reporting guidelines and was approved by The Education University of Hong Kong institutional review board. Upon giving their informed consent, respondents reported earthquake exposure (proximity to epicenter and displacement, housing destruction, physical disability, and bereavement), sociodemographics (including ethnicity, assessed in this study to reflect diversity), assets, and concerns about political destabilization.

Primary outcomes were probable depression, anxiety, and posttraumatic stress disorder (PTSD) assessed by the Patient Health Questionnaire (α = .90), Generalized Anxiety Disorder scale (α = .92), and the abbreviated PTSD Checklist (α = .89). We described the prevalence of probable psychiatric conditions (greater than validated clinical cutoffs).

Logistic regression and path analyses examined the associations between earthquake exposure and probable conditions and whether and how the associations differed across different levels of sociopolitical factors (at the .05 significance level). The eMethods in Supplement 1 provides additional information.

## Results

The 7585 respondents were mostly aged 18 to 29 years (3172 respondents [41.8%]) and 30 to 44 years (2916 respondents [38.4%]); 3613 (47.6%) were women, 1238 (16.3%) were of non-Turkish ethnicity, and 6347 (83.7%) were of Turkish ethnicity. Prevalence of current (ie, past month) probable psychiatric conditions was 44.2% for depression (3349 respondents), 31.0% for anxiety (2353 respondents), and 47.7% for PTSD (3619 respondents). The Table summarizes respondents’ sociodemographics and the peri-earthquake and sociopolitical associations of probable psychiatric conditions. Higher levels of exposure (ie, internally displaced, physical disability, or bereavement), younger age, female sex, low income, being nonmarried, Turkish ethnicity, and concerns about political destabilization were positively associated with probable conditions. Odds ratios ranged from 1.25 (95% CI, 1.11-1.42) for being nonmarried to 3.16 (95% CI, 2.13-4.69) for having a disability due to this earthquake. The Figure displays stronger associations of earthquake exposure with probable psychiatric conditions among respondents with lower compared with higher socioeconomic status (path coefficients ranged from 0.12; 95% CI, 0.01-0.24 to 0.91; 95% CI, 0.78-1.04), low compared with high assets (path coefficients ranged from 0.34; 95% CI, 0.21-0.46 to 0.89; 95% CI, 0.76-1.02), and concerns about political destabilization compared with none (path coefficients ranged from 0.46; 95% CI, 0.06-0.86 to 0.92; 95% CI, 0.78-1.06), controlling for age, sex, and ethnicity.

**Table.** Peri-Earthquake and Sociopolitical Associations of Probable Psychiatric Conditions^a^

Variables	Respondents, No. (%)	Probable^b^					
		Depression (n = 3349)		Anxiety (n = 2353)		PTSD (n = 3619)	
		OR (95% CI)	*P* value	OR (95% CI)	*P* value	OR (95% CI)	*P* value
Sociodemographics							
Age, y							
45 or above	1497 (19.74)	1 [Reference]	NA	1 [Reference]	NA	1 [Reference]	NA
30-44	2916 (38.44)	1.39 (1.22-1.58)	<.001	1.52 (1.32-1.76)	<.001	1.40 (1.23-1.59)	<.001
18-29	3172 (41.82)	2.18 (1.89-2.52)	<.001	2.28 (1.95-2.67)	<.001	2.14 (1.86-2.47)	<.001
Sex							
Male	3972 (52.37)	1 [Reference]	NA	1 [Reference]	NA	1 [Reference]	NA
Female	3613 (47.63)	1.51 (1.36-1.67)	<.001	1.48 (1.32-1.65)	<.001	1.71 (1.54-1.89)	<.001
Employment							
Employed	5579 (73.55)	1 [Reference]	NA	1 [Reference]	NA	1 [Reference]	NA
Dependent	1566 (20.65)	0.99 (0.87-1.13)	.92	0.96 (0.83-1.10)	.54	0.86 (0.79-1.02)	.10
Unemployed	440 (5.80)	1.15 (0.92-1.43)	.23	1.01 (0.79-1.27)	.97	0.82 (0.65-1.02)	.08
Monthly household income^c^							
Nonlow income	6198 (81.71)	1 [Reference]	NA	1 [Reference]	NA	1 [Reference]	NA
Low income	1387 (18.29)	1.27 (1.11-1.46)	<.001	1.34 (1.16-1.55)	<.001	1.10 (0.95-1.26)	.20
Education level							
Tertiary or above	5286 (69.69)	1 [Reference]	NA	1 [Reference]	NA	1 [Reference]	NA
Secondary or below	2299 (30.31)	0.97 (0.87-1.08)	.54	0.97 (0.86-1.09)	.59	0.95 (0.85-1.06)	.35
Marital status							
Married	4063 (53.57)	1 [Reference]	NA	1 [Reference]	NA	1 [Reference]	NA
Single, divorced, or widowed	3522 (46.43)	1.30 (1.16-1.46)	<.001	1.25 (1.11-1.42)	<.001	1.04 (0.93-1.16)	.53
Assets^d^							
High	4590 (60.51)	1 [Reference]	NA	1 [Reference]	NA	1 [Reference]	NA
Low	2995 (39.49)	1.04 (0.93-1.16)	.53	0.95 (0.84-1.07)	.36	0.93 (0.84-1.04)	.21
Ethnicity							
Non-Turkish	1238 (16.32)	1 [Reference]	NA	1 [Reference]	NA	1 [Reference]	NA
Turkish	6347 (83.68)	0.99 (0.86-1.13)	.86	1.35 (1.16-1.56)	<.001	0.99 (0.86-1.13)	.86
Earthquake exposure							
Proximity and displacement^e^							
No displacement within nonaffected provinces	6097 (80.38)	1 [Reference]	NA	1 [Reference]	NA	1 [Reference]	NA
No displacement within affected provinces	934 (12.31)	1.05 (0.90-1.22)	.54	1.08 (0.91-1.26)	.38	1.33 (1.15-1.55)	<.001
Displaced within affected provinces	331 (4.36)	1.57 (1.17-2.10)	.002	1.41 (1.05-1.89)	.02	1.98 (1.46-2.67)	<.001
Displaced from affected to nonaffected provinces	223 (2.94)	1.36 (0.94-1.97)	.11	1.69 (1.17-2.44)	.01	2.42 (1.63-3.61)	<.001
Housing destruction							
No	7214 (95.11)	1 [Reference]	NA	1 [Reference]	NA	1 [Reference]	NA
Yes	371 (4.89)	1.15 (0.84-1.58)	.39	1.27 (0.93-1.73)	.14	1.19 (0.85-1.66)	.31
Physical disability							
No disability	5747 (75.77)	1 [Reference]	NA	1 [Reference]	NA	1 [Reference]	NA
With disability not due to this earthquake	1651 (21.77)	2.36 (2.10-2.66)	<.001	2.28 (2.02-2.57)	<.001	2.32 (2.06-2.61)	<.001
With disability due to this earthquake	187 (2.47)	3.16 (2.13-4.69)	<.001	3.05 (2.13-4.36)	<.001	2.94 (1.90-4.54)	<.001
Bereavement^f^							
No loss	5991 (78.98)	1 [Reference]	NA	1 [Reference]	NA	1 [Reference]	NA
Loss of nonclose contact	1384 (18.25)	2.10 (1.84-2.40)	<.001	1.74 (1.52-1.99)	<.001	2.10 (1.84-2.41)	<.001
Loss of close contact	210 (2.77)	2.01 (1.46-2.77)	<.001	1.87 (1.37-2.55)	<.001	1.68 (1.22-2.33)	.002
Concerns about political destabilization							
No	5407 (71.29)	1 [Reference]	NA	1 [Reference]	NA	1 [Reference]	NA
Yes	2178 (28.71)	1.65 (1.48-1.83)	<.001	1.96 (1.75-2.20)	<.001	1.59 (1.42-1.77)	<.001

Abbreviations: NA, not applicable; OR, odds ratio; PTSD, posttraumatic stress disorder.

^a^ Descriptive analyses were unweighted; logistic regressions were weighted.

^b^ Probable depression was defined as a score of 10 or above on the 9-item Patient Health Questionnaire; probable anxiety was defined as a score of 10 or above on the 7-item Generalized Anxiety Disorder scale; probable PTSD was defined as a score of 14 or above on the 6-item PTSD Checklist—Specific Version.

^c^ Low monthly household income was defined as 11 499 Turkish lira (approximately US $382) or below, with reference to the net minimum wage in effect in July 2023.

^d^ Assets were defined as high (ie, high savings or property ownership) or low (ie, low savings and no property ownership). High and low savings were defined based on the cut-off of 160 000 Turkish lira (approximately US $5964) based on Turkish census data.

^e^ The affected 11 provinces were Adana, Adiyaman, Diyarbakir, Elaziğ, Gaziantep, Hatay, Kilis, Kahramanmaraş, Malatya, Osmaniye, and Şanliurfa.

^f^ Close contacts included partners, children, parents, and siblings; nonclose contacts included distant relatives, friends, and others.

**Figure.**
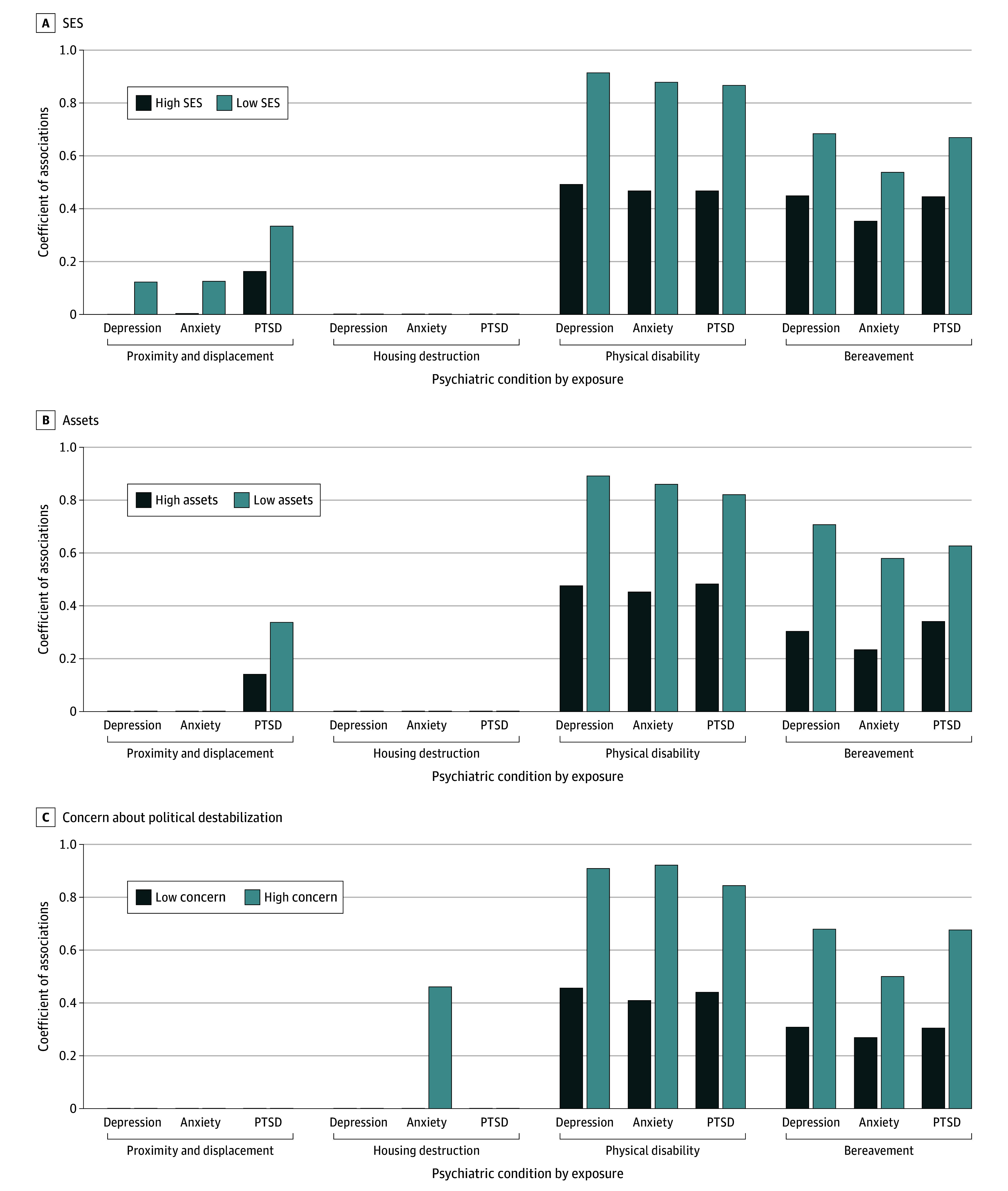
Associations Between Earthquake Exposure and Probable Psychiatric Conditions Associations are shown by level of SES, assets, and concerns about political destabilization. PTSD indicates posttraumatic stress disorder; SES, socioeconomic status.

## Discussion

This nationally representative survey reported high prevalence of current probable depression, anxiety, and PTSD (31.0%-47.7%) 7 months after the 7.8-magnitude earthquake and the political turmoil of the presidential election. Consistent with prior evidence,^3,5^ earthquake exposure was positively associated with probable psychiatric conditions. The positive earthquake-outcome associations were 2 times stronger among those with lower socioeconomic status and fewer assets, suggesting that in mass traumatic events, disadvantaged populations are more likely to experience disproportionally more adverse mental health consequences relative to those with more resources.^2,4,5^ Concerns about the ready availability of relief services^3^ might explain the observed positive association between fear of political destabilization and psychiatric conditions.^6^

The limitations of this study are the cross-sectional design and self-report measures of earthquake exposure and probable psychiatric conditions. Notwithstanding these limitations, this study highlights the role that mass traumatic events, coupled with socioeconomic and political factors, play in mental health. These observations suggest that disaster relief needs to proactively focus on populations with fewer resources and aim to restore lost resources during mass traumatic events. These findings also highlight the importance of political stability in the postdisaster context as a pathway to mitigate adverse mental health consequences of these events.

## Notes

Supplement 1.
eMethods.

eReferences.

Supplement 2.
Data Sharing Statement
