# Error in Figure

**DOI:** 10.1001/jamanetworkopen.2024.23167

**Published:** 2024-06-20

In the Research Letter titled “Sociopolitical Factors and Mental Health Following the Turkey-Syria Earthquake,”^1^ published May 15, 2024, there was an error in the Figure. The values for depression and anxiety in the proximity and displacement section of panel A should have been low socioeconomic status instead of high. This article has been corrected.^1^
