# Supplementary material for: Sociopolitical Factors and Mental Health Following the Turkey-Syria Earthquake
Source: JAMA Netw Open. 2024 May 15;7(5):e2411413. doi: 10.1001/jamanetworkopen.2024.11413 (PMC11096985; doi:10.1001/jamanetworkopen.2024.11413)
Supplement: Supplement 1. — eMethods. eReferences. [file jamanetwopen-e2411413-s001.pdf]

Supplemental Online Content

Hou WK, Tao TJ, Li CJ, et al. Sociopolitical factors and mental health following the Turkey-Syria Earthquake. *JAMA Netw Open*. 2024;7(5):e2411413. doi:10.1001/jamanetworkopen.2024.11413

eMethods.
eReferences.

This supplemental material has been provided by the authors to give readers additional information about their work.

© 2024 Hou WK et al. *JAMA Network Open.*

eMethods

Respondents and Procedures

This study involved a nationally-representative sample of Turkey (N = 7,585), recruited via the internet panel of TGM Research over the period of September to October 2023, following the reception of Ethics Committee's approval from The Education University of Hong Kong. Respondents were eligible if they were: (1) a Turkey resident, (2) 18 years of age or above, and (3) Turkish-speaking. Respondents provided written consent prior to the survey participation. All surveys were administered in Turkish, using either validated Turkish scales, or a reliable Turkish copy of the materials that underwent translation and back-translation by the research team. Attention checks were implemented, and respondents who failed to meet the required level of engagement were screened out to ensure data quality. Respondents with >5% missing data were also excluded.[1]

Detailed sampling procedure

A total of 10,110 respondents were invited. 109 (1.08%) were ineligible for inclusion and the numbers of unknown eligible was 787 (7.78%). Among 9,214 (91.14%) eligible successfully contacted panelists, 7,585 (82.32%) surveys were successfully completed online, and 1,629 (17.68%) either refused or dropped out. The final response rate was 75.91%.

Calculation formulas

Response rate = Completed / [Known eligibles + Unknown eligibles × Eligibles / (Eligibles + Ineligibles)]

Measures

Earthquake exposure and impact

Proximity to epicenter and displacement. Residential information was recorded for each respondent. We asked respondents to indicate their residential region (Aegean, Black Sea, Central Anatolia, Eastern Anatolia, Marmara, Mediterranean, Southeastern Anatolia), province (i.e., "il"), and district (i.e., "ilce"), *up to the point of the 2023 Turkey-Syria Earthquake*. Region information was then recoded into 1 = affected provinces (i.e., Adana, Adiyaman, Diyarbakir, Elaziğ, Gaziantep, Hatay, Kahramanmaraş, Kilis, Malatya, Osmaniye, and Şanliurfa) and 0 =

© 2024 Hou WK et al. *JAMA Network Open.*

non-affected provinces. Further, respondents were asked whether they were displaced (3 = *displaced* from affected to *non-affected* regions, 2 = *displaced* but still lived within *affected* regions, 1 = *not displaced* and remained within *affected* regions, 0 = *not displaced* and lived within *non-affected* regions).

Housing destruction. Respondents were asked whether their pre-earthquake residential building was destructed (1 = yes, 0 = no).

Physical disability. Respondents were asked to report whether they were diagnosed and receiving treatment for any of the disability conditions (commonly acquired under disaster contexts):[2,3] spinal cord injury (SCI), traumatic brain injury (TBI), amputation, fracture, burn, crush injury, internal organ damage, infection, mental disorders, and/or others. The respondents were also asked to specify whether any of the conditions was due to the Turkey-Syria Earthquake (1 = yes, 0 = no). The variable was then recoded into three levels: 2 = presence of physical ability due to the Turkey-Syria Earthquake, 1 = presence of physical ability not due to the earthquake, and 0 = no physical disability.

Bereavement. Respondents were asked whether they had lost anyone due to the 2023 Turkey-Syria Earthquake, which were further coded 2 = loss of a close contact (i.e., spouse or partner, child(ren), parent(s), or sibling(s)), 1 = loss of a non-close contact (i.e., distant relative(s), friend(s), or others), and 0 = no loss.

Probable psychiatric conditions

Probable depression. Depressive symptoms were assessed using the Turkish version of the 9-item Patient Health Questionnaire (PHQ-9).[4,5] Respondents rated the frequency of their experiencing depressive symptoms *over the past two weeks* (0 = not at all, 1 = on several days, 2 = on more than half of the days, 3 = nearly every day). Summed scores were generated (range = 0–27), with higher scores indicating higher depressive symptoms. Probable depression was defined as a score of 10 or above.[6] The scale was found to have high internal consistency among Turkish populations (α = .842).[4] The internal consistency in the current study was high (α = .899).

Probable anxiety. The Turkish version of the 7-item Generalized Anxiety Disorder scale (GAD-7) was used to assess anxiety symptoms.[7,8] Respondents rated the frequency of their experiencing anxiety symptoms *over the past two weeks* (0 = not at all, 1 = on several days, 2 = on more than half of the days, 3 = nearly every day). Higher summed scores (range = 0–21) indicated higher anxiety symptoms. Probable anxiety was defined as a score of 10 or

© 2024 Hou WK et al. *JAMA Network Open.*

above.[9] The scale demonstrated high validity, reliability and good psychometric features within the Turkish sample.[7] The internal consistency in the current study was high (α = .921).

Probable posttraumatic stress disorder (PTSD). PTSD symptoms related to the 2023 Turkey-Syria Earthquake were assessed using the Turkish version of the PTSD Checklist – 6-item Specific Version (PCL-6-S).[10,11] Each symptom was evaluated with a 5-point scale (1 = not at all, 5 = extremely). Higher scores (range = 6–30) indicated greater PTSD symptoms. Probable PTSD was defined as a score of 14 or above.[12] Good internal consistency, validity, and diagnostical performance were established in this previous study.[10] The internal consistency in the current study was high (α = .889).

Sociodemographic profile

A standardized proforma was used to obtain demographic information, including age, gender, marital status, education level, employment status, monthly household income (5,749 TL or below, 5,750−11,499 TL, 11,500−22,999 TL, 23,000−34,499 TL, 34,500−45,999 TL, 46,000−57,499 TL, 57,500−68,999 TL; 69,000−80,499 TL, 80,500−91,999 TL, 92,000−103,499 TL, 103,500−114,999 TL, 115,000 TL or above; with reference to the net minimum wage of 11,402 TL in effect in July 2023, income was recoded into low-income (11,499 TL or below), mid-income (11,500−22,999 TL), and high-income (23,000 TL or above)),[13] and ethnicity.

For the purpose of this study, a composite score for socioeconomic status (SES) was collectively defined based on marital status (0 = married, 1 = non-married), education level (0 = tertiary or above, 1 = secondary or below), and monthly household income (0 = non-low, 1 = low).[14] The composite score was then recoded into 1 = low SES (an original composite score of 1 or above) and 0 = high SES (an original composite score of 0), with reference to similar research.[15,16]

Liquid and physical assets

Respondents were asked to report their personal savings (no savings, 79,999 TL or below, 80,000–159,999 TL, 160,000–319,999 TL, 320,000–479,999 TL, 480,000–639,999 TL, 640,000–799,999 TL, 800,000–999,999 TL, 1,000,000 TL or above). They were reminded to take into consideration all types of accounts, including cash, savings, checking accounts, stocks, bonds, mutual funds, retirement funds (such as pensions, IRAs, 401Ks, etc.), and certificates of deposit. The amount of 160,000 TL (approximately 5,964 USD) was a cut-off line for median personal

© 2024 Hou WK et al. *JAMA Network Open.*

wealth based on Turkish census data.[17] Respondents were asked to report property ownership (1 = no, 0 = yes). Assets were recoded as 1 = low (i.e., low savings and no property ownership) and 0 = high (i.e., high savings and/or property ownership) on the basis of previous research.[18,19]

Concern about political destabilization

Adapted from previous research,[20,21] respondents' concern about political destabilization was assessed using an item on perceived threat from the opposition on the union of Turkey on a 6-point scale (1 = strongly oppose, 2 = oppose, 3 = somewhat oppose, 4 = somewhat agree, 5 = agree, 6 = strongly agree). The scores were then binary split into 1 = yes (i.e., somewhat agree to strongly agree) and 0 = no (i.e., somewhat oppose to strongly oppose).

Data Analysis

Missing data (<1%) were handled by multiple imputation with the predictive mean matching (PMM) approach with a total of 10 imputed datasets.

First, binary logistic regression analysis examined the association between peri-earthquake and socio-political factors and probable psychiatric conditions. These factors included earthquake exposure and impact (proximity to epicenter and displacement, housing destruction, physical disability, loss of loved one(s)), demographics, socioeconomic status, assets, and concern about political destabilization.

Next, to test how the earthquake-disorder association was moderated by socio-political factors, three sets of path analyses were conducted in M*plus* followed by simple slopes analyses. Moderators included socioeconomic status (higher vs. lower), assets (high vs. low), and concern about political destabilization (yes vs. no). In each set of path model, independent variables included the four variables on earthquake exposure/impact, the moderator, and four interaction terms of each earthquake variable and moderator, whereas dependent variables included the three probable mental disorder variables. Demographic covariates (age, gender, ethnicity) were adjusted for. Simple slopes tests were conducted to investigate differences in the earthquake-disorder association, under the different levels of the socio-political determinants.

© 2024 Hou WK et al. *JAMA Network Open.*

The weighted least square mean and variance adjusted (WLSMV) estimator was used. Data-model fit for the path analyses was holistically assessed with the Comparative Fit Index (CFI), Tucker-Lewis Index (TLI), root mean square error of approximation (RMSEA), and standardized root mean square residual (SRMR). The model was considered reliable with CFI and TLI indices >0.90 and RMSEA and SRMR indices <0.08.[22]

eReferences

1. Jakobsen JC, Gluud C, Wetterslev J, Winkel P. When and how should multiple imputation be used for handling missing data in randomised clinical trials – a practical guide with flowcharts. *BMC Med Res Methodol*. 2017;17:162. https://doi.org/10.1186/s12874-017-0442-1
2. Reinhardt JD, Zhang X, Van Dyke C, Ehrmann C, Li L, Zhao Z, Zhou M, Li H. Post-traumatic stress disorder in a population of 2008 Wenchuan earthquake survivors with disabilities: the role of environmental barriers. *Disabil Rehabil*. 2021;43(19):2720–2728. https://doi.org/10.1080/09638288.2020.1714756
3. Tang B, Chen Q, Chen X, Glik D, Liu X, Liu Y, Zhang L. Earthquake-related injuries among survivors: a systematic review and quantitative synthesis of the literature. *Int J Disaster Risk Reduct*. 2017;21:159–167. https://doi.org/10.1016/j.ijdrr.2016.12.003
4. Sari YE, Kokoglu B, Balcioglu H, Bilge U, Colak E, Unluoglu I. Turkish reliability of the Patient Health Questionnaire-9. *Biomed Res-India*. 2016;27:S460–S462.
5. Kroenke K, Spitzer RL, Williams JB. The PHQ-9: validity of a brief depression severity measure. *J Gen Intern Med*. 2001;16(9):606–613. https://doi.org/10.1046/j.1525-1497.2001.016009606.x
6. Levis B, Benedetti A, Thombs BD. Accuracy of Patient Health Questionnaire-9 (PHQ-9) for screening to detect major depression: individual participant data meta-analysis. *BMJ*. 2019;365:I1476. https://doi.org/10.1136/bmj.l1476
7. Konkan R, Şenormanci Ö, Güçlü O, Aydin E, Sungur MZ. Validity and reliability study for the Turkish adaptation of the Generalized Anxiety Disorder-7 (GAD-7) scale. *Arch Neuropsychiatry*. 2013;50:53–58. https://doi.org/10.4274/npa.y6308
8. Spitzer RL, Kroenke K, Williams JB, Löwe B. A brief measure for assessing generalized anxiety disorder: the GAD-7. *Arch Intern Med*. 2006;166(10):1092–1097. https://doi.org/10.1001/archinte.166.10.1092

© 2024 Hou WK et al. *JAMA Network Open.*

9. Plummer F, Manea L, Trepel D, McMillan D. Screening for anxiety disorders with the GAD-7 and GAD-2: a systematic review and diagnostic metaanalysis. *Gen Hosp Psychiatry*. 2016;39:24–31. https://doi.org/10.1016/j.genhosppsych.2015.11.005
10. Kocabaşoğlu N, Özdemir AÇ, Yargıç İ, Geyran P. Türkçe "PTSD Checklist - Civilian Version" (PCL-C) Ölçeğinin Geçerlilik ve Güvenilirliği [The validity and safety of Turkish "PTSD Checklist – Civilian Version" (PCL-C) Scale]. *Yeni Symposium*. 2005;43(3):126–134.
11. Lang AJ, Stein MB. An abbreviated PTSD checklist for use as a screening instrument in primary care. *Behav Res Ther*. 2005;43:585–594. https://doi.org/10.1016/j.brat.2004.04.005
12. Lang AJ, Wilkins K, Roy-Byrne PP, Golinelli D, Chavira D, Sherbourne C, Rose RD, Bystritsky A, Sullivan G, Craske MG, Stein MB. Abbreviated PTSD Checklist (PCL) as a guide to clinical response. *Gen Hosp Psychaitry*. 2012;34(4)332–338. https://doi.org/10.1016/j.genhosppsych.2012.02.003
13. Dalgiç-tetikol DE, Köksal E, Güloğlu B. The evolution of the digital divide in Turkey. *J Res Econ*. 2023;7(1):65–83. http://dx.doi.org/10.29228/JORE.24
14. Ettman CK, Cohen GH, Abdalla SM, Trinquart L, Castrucci BC, Bork RH, Clark MA, Wilson IB, Vivier PM, Galea S. Assets, stressors, and symptoms of persistent depression over the first year of the COVID-19 pandemic. *Sci Adv*. 2022;8(9):eabm9737. https://doi.org/10.1126/sciadv.abm9737
15. Sacre H, Haddad C, Hajj A, Zeenny RM, Akel M, Salameh P. Development and validation of the Socioeconomic Status Composite Scale (SES-C). *BMC Public Health*. 2023;23(1):1619. https://doi.org/10.1186/s12889-023-16531-9
16. Wah W, Earnest A, Sabanayagam C, Cheng C-Y, Ong MEH, Wong TY, Lamoureux EL. Composite measures of individual and area-level socio-economic status are associated with visual impairment in Singapore. *PLoS One*. 2015;10(11):e0142302. http://doi.org/10.1371/journal.pone.0142302
17. Credit Suisse. Global wealth databook 2022. 2022. https://www.credit-suisse.com/about-us/en/reports-research/global-wealth-report.html
18. Ettman CK, Abdalla SM, Cohen GH, Sampson L, Vivier PM, Galea, S. Low assets and financial stressors associated with higher depression during COVID-19 in a nationally representative sample of US adults. *J Epidemiol Community Health*. 2020;75(6):501–508. http://dx.doi.org/10.1136/jech-2020-215213

© 2024 Hou WK et al. *JAMA Network Open.*

19. Hou WK, Lee TM, Liang L, Li TW, Liu H, Ettman CK, Galea S. Civil unrest, COVID-19 stressors, anxiety, and depression in the acute phase of the pandemic: a population-based study in Hong Kong. *Soc Psychiatry Psychiatr Epidemiol*. 2021;56:1499–1508. https://doi.org/10.1007/s00127-021-02037-5

20. Hou WK, Canetti D, Ma TW, Hall BJ, Lau KM, Ng SM, Hobfoll SE. (2018). What predicts threat perceptions toward people opposing to the government? a population-based study following Umbrella Movement, Hong Kong. *J Soc Political Psychol*. 2018;6(2):383–400. https://doi.org/10.5964/jspp.v6i2.791

21. Shamir M, Sullivan JL. Jews and Arabs in Israel: everybody hates somebody, sometime. *J Confl Resolut*. 1985;29(2):283–305. https://doi.org/10.1177/0022002785029002006

22. Hooper D, Coughlan J, Mullen M. Structural equation modelling: guidelines for determining model fit. *Electron J Bus Res Methods*. 2008;6(1):53–60.

© 2024 Hou WK et al. *JAMA Network Open.*
