# Supplementary material for: Sociopolitical Factors and Mental Health Following the Turkey-Syria Earthquake
Source: JAMA Netw Open. 2024 May 15;7(5):e2411413. doi: 10.1001/jamanetworkopen.2024.11413 (PMC11096985; doi:10.1001/jamanetworkopen.2024.11413)
Supplement: Supplement 2. — Data Sharing Statement [file jamanetwopen-e2411413-s002.pdf]

Data Sharing Statement

Hou. Sociopolitical Factors and Mental Health Following the Turkey-Syria Earthquake. *JAMA Netw Open*. Published May 15, 2024. doi:10.1001/jamanetworkopen.2024.11413

Data
Data available: No

Additional Information
Explanation for why data not available: The data of the study are available from the corresponding author upon reasonable request.
